# Recombinant *Beauveria bassiana* expressing *Bacillus thuringiensis* toxin Cyt1Aa: a promising approach for enhancing *Aedes* mosquito control

**DOI:** 10.1128/spectrum.03792-23

**Published:** 2024-05-29

**Authors:** Sheng-Qun Deng, Ni Li, Xu-Ke Yang, Hong-Zheng Lu, Jia-Hua Liu, Zhe-Yu Peng, Lin-Min Wang, Mao Zhang, Chao Zhang, Chen Chen

**Affiliations:** 1 Department of Pathogen Biology, School of Basic Medical Sciences, Anhui Medical University, Hefei, China; 2 Anhui Province Key Laboratory of Zoonoses, the Key Laboratory of Zoonoses of High Institutions in Anhui, Anhui Medical University, Hefei, China; 3 Department of Microbiology, School of Basic Medical Sciences, Anhui Medical University, Hefei, China; Hubei University of Medicine, Hubei Province, Shiyan, China

**Keywords:** *Beauveria bassiana*, Cyt1Aa, *Aedes aegypti*, *Aedes albopictus*, mosquito control

## Abstract

The entomopathogenic fungus *Beauveria bassiana* provides an eco-friendly substitute to chemical insecticides for mosquito control. Nevertheless, its widespread application has been hindered by its comparatively slow efficacy in eliminating mosquitoes. To augment the potency of *B. bassiana* against *Aedes* mosquitoes, a novel recombinant strain, *Bb*-Cyt1Aa, was developed by incorporating the *Bacillus thuringiensis* toxin gene *Cyt1Aa* into *B. bassiana*. The virulence of *Bb*-Cyt1Aa was evaluated against *Aedes aegypti* and *Aedes albopictus* using insect bioassays. Compared to the wild-type (WT) strain, the median lethal time (LT_50_) for *A. aegypti* larvae infected with *Bb*-Cyt1Aa decreased by 33.3% at a concentration of 1 × 10^8^ conidia/mL and by 22.2% at 1 × 10^7^ conidia/mL. The LT_50_ for *A. aegypti* adults infected with *Bb*-Cyt1Aa through conidia ingestion was reduced by 37.5% at 1 × 10^8^ conidia/mL and by 33.3% at 1 × 10^7^ conidia/mL. Likewise, the LT_50_ for *A. aegypti* adults infected with *Bb*-Cyt1Aa through cuticle contact decreased by 33.3% and 30.8% at the same concentrations, respectively. Furthermore, the *Bb*-Cyt1Aa strain also demonstrated increased toxicity against both larval and adult *A. albopictus*, when compared to the WT strain. In conclusion, our study demonstrated that the expression of *B. thuringiensis* toxin Cyt1Aa in *B. bassiana* enhanced its virulence against *Aedes* mosquitoes. This suggests that *B. bassiana* expressing Cyt1Aa has potential value for use in mosquito control.

**IMPORTANCE:**

*Beauveria bassiana* is a naturally occurring fungus that can be utilized as a bioinsecticide against mosquitoes. Cyt1Aa is a delta-endotoxin protein produced by *Bacillus thuringiensis* that exhibits specific and potent insecticidal activity against mosquitoes. In our study, the expression of this toxin Cyt1Aa in *B. bassiana* enhances the virulence of *B. bassiana* against *Aedes aegypti* and *Aedes albopictus*, thereby increasing their effectiveness in killing mosquitoes. This novel strain can be used alongside chemical insecticides to reduce dependence on harmful chemicals, thereby minimizing negative impacts on the environment and human health. Additionally, the potential resistance of *B. bassiana* against mosquitoes in the future could be overcome by acquiring novel combinations of exogenous toxin genes. The presence of *B. bassiana* that expresses Cyt1Aa is of significant importance in mosquito control as it enhances genetic diversity, creates novel virulent strains, and contributes to the development of safer and more sustainable methods of mosquito control.

## INTRODUCTION

*Aedes* mosquitoes have emerged as vectors of pathogens for many dangerous diseases, such as dengue fever and Zika virus disease (1). The alarming prevalence of these diseases, with staggering hundreds of millions of cases reported, has made it essential to address and control the population of *Aedes* mosquitoes (2). While traditional control methods for *Aedes* mosquitoes, such as chemical insecticides, have proven effective, their persistent use has resulted in an increase in mosquitoes’ resistance to them (3–5). This resistance means that higher doses of the chemical insecticide may be needed to kill mosquitoes, eventually rendering it completely ineffective (6, 7). It is crucial to adopt alternative or supplemental approaches for *Aedes* mosquito control (8).

*B. bassiana* is a naturally occurring fungus that can be used as a biological insecticide against mosquitoes (9–14). It has a multistep process to kill mosquitoes (15). Conidia attach to the mosquito cuticle, germinating and penetrating the cuticle and invading and proliferating in the hemocoel (16, 17). Once in the hemocoel, hyphal bodies secrete many toxic secondary metabolites, suppressing the host immune system (18, 19). Eventually, *Aedes* mosquitoes die through a combination of immune system evasion and nutrient deprivation (20, 21). These multiple actions make it difficult for mosquitoes to develop resistance mechanisms, as mosquitoes need to adapt to multiple targets simultaneously. Unlike chemical insecticides, which have a broad range of activity and can harm nontarget organisms, it is a highly selective control method for particular pests (22). By selectively targeting, it minimizes environmental impact and helps maintain ecological balance. *B. bassiana* can be a potential alternative to chemical insecticides for mosquito control in the future.

While *B. bassiana* has shown promise as a biological insecticide for *Aedes* mosquito control, its relatively slow action in killing mosquitoes, compared with chemical insecticides, has impeded its widespread application (23). Further research is needed to overcome these challenges to increase the virulence of *B. bassiana* against mosquitoes. Genetic modification techniques can be employed to introduce specific traits or genes into *B. bassiana*, which enhances its virulence (24, 25). For example, genes encoding toxic proteins, such as *Bacillus thuringiensis* toxins, can be inserted into the *B. bassiana* genome to increase its pathogenicity against target mosquitoes (26). Cyt1Aa is an exogenous toxin produced by *B. thuringiensis* that displays specific and potent insecticidal activity against mosquitoes (27). It targets the *Aedes* mosquito midgut, breaking down the midgut cells and ultimately resulting in the death of mosquitoes (28). In this study, *B. bassiana* was modified to express the *B. thuringiensis* toxin Cyt1Aa to increase its virulence through genetic modification. The virulence of *B. bassiana* expressing Cyt1Aa (*Bb*-Cyt1Aa) was evaluated against *Aedes albopictus* and *Aedes aegypti* at three concentrations using insect bioassays. The results demonstrated that *Bb*-Cyt1Aa significantly reduced the survival of *A. albopictus* and *A. aegypti* compared to the wild-type (WT) strain, suggesting that expressing Cyt1Aa in *B. bassiana* has great potential for mosquito control.

## MATERIALS AND METHODS

### Mosquitoes

The Foshan strain of *A. albopictus* and the Zhanjiang strain of *A. aegypti* were obtained from Guangdong Provincial Center for Disease Control and Prevention, China. All mosquitoes were kept at 28 ± 1°C, 80 ± 5% relative humidity (RH), and 14 h light:10 h dark photoperiods. Mosquito larvae were fed turtle food (Inch-Gold Fish Food Co., Ltd., Shenzhen, China). Mosquito adults were fed 10% glucose solution every day. Adult female mosquitoes were fed with Kunming mouse blood, which was provided by the Animal Experiment Center of Anhui Medical University, in order to induce egg-laying. The use of animals was approved by the Experimental Animal Ethics Committee of Anhui Medical University (approval code: LLSC20210773).

### Microbial strains and media

The *B. bassiana* GIM3.428 strain (wild type) was purchased from the Guangdong Microbiology Culture Center and maintained on potato dextrose agar (PDA) at 4°C for preservation and at 25°C for colony growth. The wild-type strain was grown at 25℃ for 2 days in Sabouraud dextrose broth (SDB) and then transferred (5 mL) into 50 mL of new glucose mineral (GM) for further incubation at 25°C for 2 days. The PDA contained 20% (wt/vol) potato powder, 2% (wt/vol) glucose, 2% (wt/vol) agar, 0.5% (wt/vol) tryptone, 0.3% (wt/vol) KH_2_PO_4_, and 0.15% (wt/vol) MgSO_4_. The SDB contained 4% (wt/vol) glucose, 1% (wt/vol) tryptone, and 1% (wt/vol) yeast extract powder. The new GM contained 4% (wt/vol) glucose, 2% (wt/vol) peanut powder, 0.3% (wt/vol) KH_2_PO_4_, and 0.03% (wt/vol) MgSO_4_.

### Gene synthesis and vector construction

The coding sequence of Cyt1Aa (GenBank: MT995838.1) was synthesized with the *B. bassiana* preferred coding usage (Tsingke Biotech Co., Ltd., Beijing, China) and cloned between the *Eco*RI and *Xho*I sites of pBARGPE1 to generate the plasmid pBARGPE1-Cyt1Aa. This plasmid retains a strong gpdA promoter to drive the insert’s gene expression and resistance gene (Bar) to express the selectable marker that would provide resistance to phosphinothricin (PPT).

### Fungal transformation

The plasmid pBARGPE1-Cyt1Aa was linearized with *Pvu*I (Takara, Beijing, China). The *B. bassiana* blastospores were collected and counted to 6 × 10^6^ conidia/mL. The mixture containing 1 µg linearized pBARGPE1-Cyt1Aa and 6 × 10^6^ conidia/mL blastospores in a final sample volume of 100 µL was subjected to electroporation for 5 ms at 20 KV/cm in a 0.2 cm-gap MicroPulser electroporation cuvette (Bio-Rad, Hercules, California, USA). The transformants were grown on PDA plates containing 200 µg/mL PPT for 7–10 days at 25°C. After single spore isolation and subsequent subculturing for three generations on PDA with 200 µg/mL PPT at 25°C, the genomic DNAs of three generations were extracted and used as polymerase chain reaction (PCR) templates. To verify the presence of the *Cyt1Aa* gene in the *B. bassiana* genome, PCR was performed using the primer pairs *Cyt1Aa* gene (Cyt-F ATGGAAAATTTAAATCATTG, and Cyt-R, AATCTCCCAAGGTAATTATC), *Bar* gene (Bar-F, ATGAGCCCAGAACGACGCCC and Bar-R, GATCTCGGTGACGGGCAGGAC), and 18s gene (18s-F, AGGGCTCTTTTGGGTCTTG and 18s-R, GTTTCAGCCTTGCGACCAT). Furthermore, to verify the mitotic stability of their PPT resistance, the recombinants were subcultured for three generations on PDA without PPT at 25°C. The recombinants were analyzed by PCR. A stable recombinant strain named *Bb*-Cyt1Aa was selected for subsequent experiments.

### Identification of Cyt1Aa expression in *Bb*-Cyt1Aa

The WT and *Bb*-Cyt1Aa strains were grown on PDA for 7 days. The conidia were collected using a scraper and placed into an Eppendorf (EP) tube containing ddH_2_O. To verify the transcription of the *Cyt1Aa* gene, total RNA was extracted from the WT and *Bb*-Cyt1Aa strains and used as reverse transcription-PCR (RT‒PCR) templates. RT‒PCR was performed using the primer pairs of the *Cyt1Aa* gene and 18S gene. Cyt1Aa polyclonal antibodies were raised in rabbits (Tsingke Biotech Co., Ltd., Beijing, China). To verify the translation of the *Cyt1Aa* gene in *Bb*-Cyt1Aa, the total proteins were extracted from the PDA culture supernatant. Fifty micrograms of Cyt1Aa protein extracts were analyzed by SDS‒PAGE with 15% acrylamide, and western blotting was then performed.

### Survival assays

The *B. bassiana* strains were grown and maintained on PDA at 25°C. Conidial suspensions in 0.02% (vol/vol) Tween 80 were prepared from 7-day-old cultures. The virulence of the *Bb*-Cyt1Aa and WT strains was compared by conducting three survival assays of *A. albopictus* and *A. aegypti* that were exposed to *Bb*-Cyt1Aa and WT conidial suspensions at three different concentrations (i.e., 10^8^, 10^7^, and 10^6^ conidia/mL). Three survival assays were performed at 28 ± 2°C with 80 ± 5% RH and a 14 h light:10 h dark photoperiod.

The second-instar *Aedes* larvae (*n* = 30 per treatment) were treated with 50 mL ddH_2_O-diluted conidial suspensions of 1 × 10^6^ conidia/mL (low), 1 × 10^7^ conidia/mL (middle), and 1 × 10^8^ conidia/mL (high) of *B. bassiana*. Thirty individuals in ddH_2_O without fungi were used as the control. The larvae were provided with turtle food at a rate of 0.2–0.3 mg/larva per day. The survival of the larvae was observed every 12 h for 2 weeks. The insects reaching the pupal stage were treated as censored data.

Adult female *Aedes* mosquitoes (*n* = 30 per treatment) were infected by feeding with 10% glucose solution containing *Bb*-Cyt1Aa and WT strains. The conidia had different concentrations of 1 × 10^6^ conidia/mL (low), 1 × 10^7^ conidia/mL (medium), and 1 × 10^8^ conidia/mL (high), and the control group was fed pure 10% glucose solution.

The adult female *Aedes* mosquitoes were transferred to the filter (placed on a 300 mL plastic cup covered with a net), which had absorbed 10 mL of conidial suspension at 1 × 10^6^ conidia/mL (low), 1 × 10^7^ conidia/mL (middle), or 1 × 10^8^ conidia/mL (high), or 0.02% Tween 80 (control) for 3 h. Then, the infected or control adults were transferred separately to different plastic cups (30 mosquitoes/cup) and fed with 10% glucose solution once a day. The dead adult mosquitoes in each treatment group were counted and removed every 12 h until the last mosquito died.

Each treatment described above was repeated three times, and the entire experiment was performed thrice. The Kaplan–Meier analysis and log-rank tests were utilized to ascertain the variation in survival rates among the different groups. Significance was defined by *P* < 0.05.

### Dissection and paraffin sections

To verify the effect of Cyt1Aa on the mosquito midgut, we infected *A. aegypti* with *Bb*-Cyt1Aa and *Bb*-WT, dissected the midgut, and prepared paraffin sections. *A. aegypti* infected with ddH_2_O was chosen as the control group. *A. aegypti* abdomens were taken as the samples of paraffin sections and fixed using 4% paraformaldehyde. Then, it was infiltrated with molten paraffin wax and embedded in a mold. After being sectioned at the largest diameter part using a Leica RM2235 microtome (Leica, Wetzlar, Germany), thin sections were mounted on glass slides and stained with hematoxylin and eosin. The section size of the paraffin sections was 5 μm. Finally, the prepared paraffin sections were examined under a microscope to observe the midgut structure and identify abnormalities.

## RESULTS

### *B. thuringiensis* toxin Cyt1Aa was expressed in *Beauveria bassiana*

**FIG 1:**
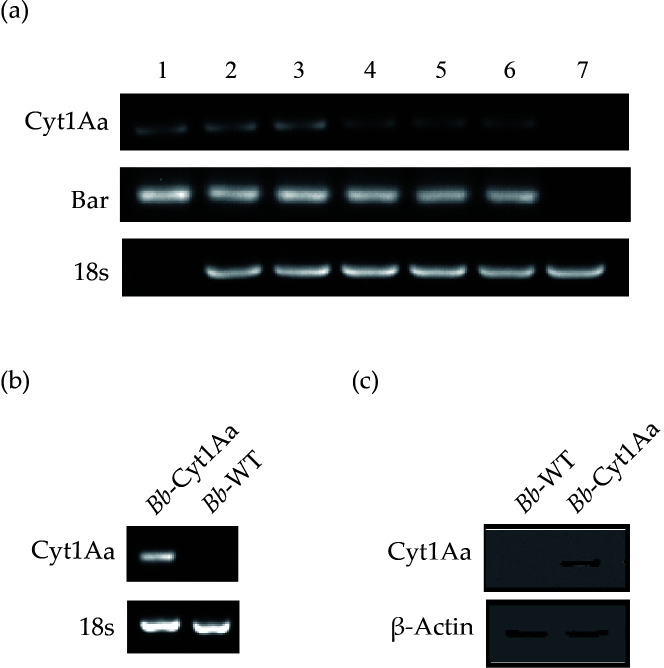
Evidence for Cyt1Aa expression in the *Bb*-Cyt1Aa strain. (a) Conventional PCR products were amplified with the DNA of the plasmid pBARGPE1-Cyt1Aa (Line 1) and the genomic DNAs of *Bb*-Cyt1Aa (Lines 2–6) and *Bb*-WT (Line 7). Line 1, the plasmid pBARGPE1-Cyt1Aa. Lines 2–4, the recombinants that were subcultured for three generations on PPT+medium with positive results. Line 5, the recombinant that was subcultured for one generation on PDA medium with a positive result. Line 6, the recombinant that was subcultured for one generation on PPT+medium with positive result. (b) RT-PCR detection of Cyt1Aa (750 bp) transcription in *Bb*-Cyt1Aa with positive results and in the *Bb*-WT control with negative results. 18S rRNA was detected in both *Bb*-Cyt1Aa and *Bb*-WT strains. (c) Western blot detection of Cyt1Aa (27.3 kDa) expression in the *Bb*-WT control with negative results and in the *Bb*-Cyt1Aa strain with positive results. The β-actin protein (43 kDa) was detected in both the *Bb*-Cyt1Aa and *Bb*-WT strains.

The competent spores of *B. bassiana* were successfully transformed with linear plasmid pBARGPE1-Cyt1Aa, resulting in the production of a single transgenic on the PDA plate with a concentration of 200 µg/mL PPT (PPT+). The recombinant strain was capable of culturing on 200 µg/mL PPT+plates for three generations. After being transplanted to PPT-free PDA plates, it grew normally for three generations. Consequently, it was transplanted to a 500 µg/mL PPT+plate, where continuous growth was observed. There were no variations in appearance between the recombinant and WT strains. The agarose gel exhibited the target fragments from *Bb*-Cyt1Aa samples, while no fragments were in the WT, suggesting that the Cyt1A gene was consistently inherited in the *Bb*-Cyt1Aa genome (Fig. 1a). RT-PCR and western blotting detected the expression of Cyt1Aa in the recombinant strain, while no expression of Cyt1Aa was detected in the WT strain (Fig. 1b and c).

### The survival of *A. aegypti* was reduced by *Bb-*Cyt1Aa infection compared with *Bb-*WT infection

**FIG 2:**
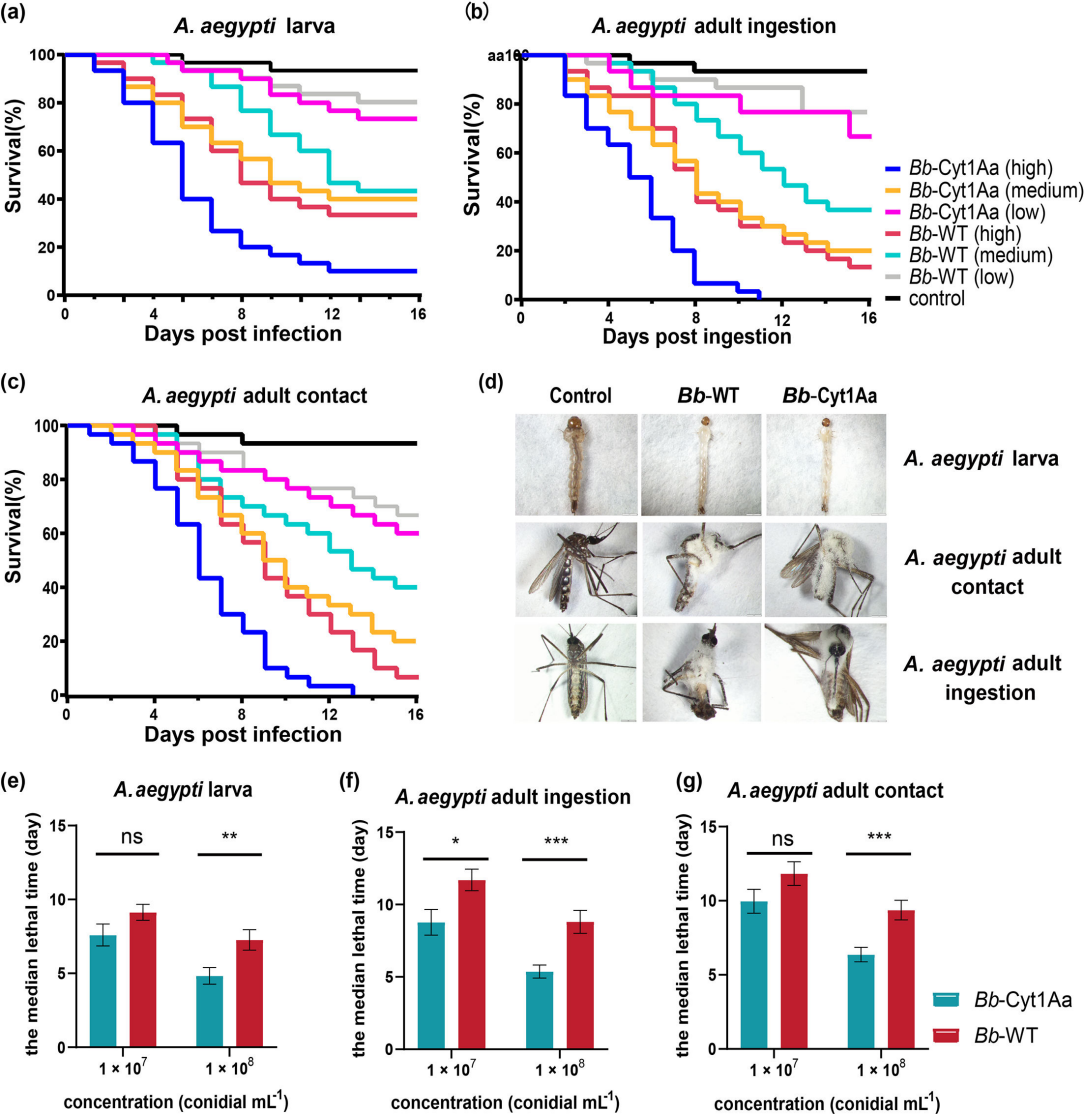
Insecticidal activity of *Bb*-Cyt1Aa and WT strain against *A. aegypti* mosquitoes. (a) Survival rates of *A. aegypti* larvae infected with 1 × 10^8^ (high), 1 × 10^7^ (medium), and 1 × 10^6^ (low) conidia mL^−1^ suspensions of *Bb*-Cyt1Aa and WT. (b and c) Survival rates of *A. aegypti* adults infected with (high) 1 × 10^8^, (medium) 1 × 10^7^, and (low) 1 × 10^6^ conidia mL^−1^ suspensions of *Bb*-Cyt1Aa and WT via conidia ingestion (b) or cuticle contact (c). (d) Symptoms of *A. aegypti* (larvae or adults) 6 days after infection. *A aegypti* infected *Bb*-Cyt1Aa died more quickly compared to that infected WT. *Bb*-Cyt1Aa exhibited a higher quantity and strength of hyphae growth on the surface of *A. aegypti* adults compared to WT. (e) The median lethal times (LT_50_s) of *Bb*-Cyt1Aa and WT against *A. aegypti* larvae at 1 × 10^8^ and 1 × 10^7^ conidia/mL. (f and g) LT_50_s of *Bb*-Cyt1Aa and WT against *A. aegypti* adults at 1 × 10^8^ and 1 × 10^7^ conidia/mL via conidia ingestion (f) or cuticle contact (g). Data are presented as the median ± SEM (*P < 0.05, **P < 0.01, ***P < 0.001).

In insect bioassays, statistically significant differences were observed in the mortalities between *Bb*-Cyt1Aa and WT strains against larval or adult *A. aegypti* (Table 1). The mortality rates of *A. aegypti* infected with *Bb*-Cyt1Aa and WT strains gradually increased with prolonged time and increased concentration (Fig. 2a through c). *B. bassiana* hyphae grew on the surface of mosquitoes via conidium ingestion or contact (Fig. 2d). There was a statistically significant difference in survival among the *Bb*-Cyt1Aa or WT strains at three given concentrations (Table S1). Statistically significant differences were found between *Bb*-Cyt1Aa and WT strains at 10^8^ and 10^7^ conidia/mL (Table S2). The LT_50_s of *A. aegypti* infected with *Bb*-Cyt1Aa through conidia ingestion or through cuticle contact were reduced compared with those treated with the WT at 10^8^ and 10^7^ conidia/mL (Fig. 2e through g; Table 2).

**TABLE 1:** Results of the log-rank test for the *Bb*-Cyt1Aa and WT strains against *A. albopictus* or *A. aegypti* mosquitoes (larvae or adults)^*a*^

Mosquitoes	*χ* ^2^	Df	*P*
*A. aegypti* larvae	4.067	1	0.044
*A. aegypti* adults (ingestion)	7.015	1	0.008
*A. aegypti* adults (contact)	4.660	1	0.031
*A. albopictus* larvae	4.210	1	0.040
*A. albopictus* adults (ingestion)	7.212	1	0.007
*A. albopictus* adults (contact)	6.213	1	0.013

^*a*^ *P* < 0.05 means that the difference is significant.

**TABLE 2:** The median lethal times (LT_50_s) of the *Bb*-Cyt1Aa and WT strains against *A. aegypti* and *A. albopictus* mosquitoes (larvae or adults)

Mosquitoes	Fungal strains	LT_50_ (Median ± standard error), day	
	(conidia/mL)	1 × 10^8^	1 × 10^7^
*A. aegypti* larvae	*Bb-*Cyt1Aa	4.0 (±0.4)	7.0 (±1.4)
	*Bb*-WT	6.0 (±0.9)	9.0 (±1.1)
*A. aegypti* adults (ingestion)	*Bb-*Cyt1Aa	5.0 (±0.6)	8.0 (±0.7)
	*Bb*-WT	8.0 (±0.6)	12.0 (±1.4)
*A. aegypti* adults (contact)	*Bb-*Cyt1Aa	6.0 (±0.5)	9.0 (±0.9)
	*Bb*-WT	9.0 (±0.7)	13.0 (±1.6)
*A. albopictus* larvae	*Bb-*Cyt1Aa	4.0 (±0.5)	7.0 (±0.6)
	*Bb*-WT	7.0 (±0.7)	9.0 (±1.1)
*A. albopictus* adults (ingestion)	*Bb-*Cyt1Aa	5.0 (±0.8)	7.0 (±1.1)
	*Bb*-WT	8.0 (±0.9)	11.0 (±1.6)
*A. albopictus* adults (contact)	*Bb-*Cyt1Aa	5.0 (±0.5)	8.0 (±0.9)
	*Bb*-WT	8.0 (±0.8)	12.0 (±1.4)

In comparison with the results for WT, the LT_50_ of *Bb*-Cyt1Aa for *A. aegypti* larvae declined by 33.3% at a concentration of 10^8^ conidia/mL and 22.2% at 10^7^ conidia/mL. Compared to the WT strain, the LT_50_ of *Bb*-Cyt1Aa for *A*. *aegypti* infected through conidia ingestion declined by 37.5% at 10^8^ conidia/mL and 33.3% at 10^7^ conidia/mL, respectively, and those for *A*. *aegypti* infected through cuticle contact declined by 33.3% and 30.8% at the same concentrations, respectively. The bioassays of *A aegypti* demonstrated that *Bb*-Cyt1Aa increased the virulence against *A. aegypti*, resulting in 1.29- to 1.6-fold higher toxicity compared to that of the WT strain.

### The midgut of *A. aegypti* can be a route of *Bb-*Cyt1Aa infection

**FIG 3:**
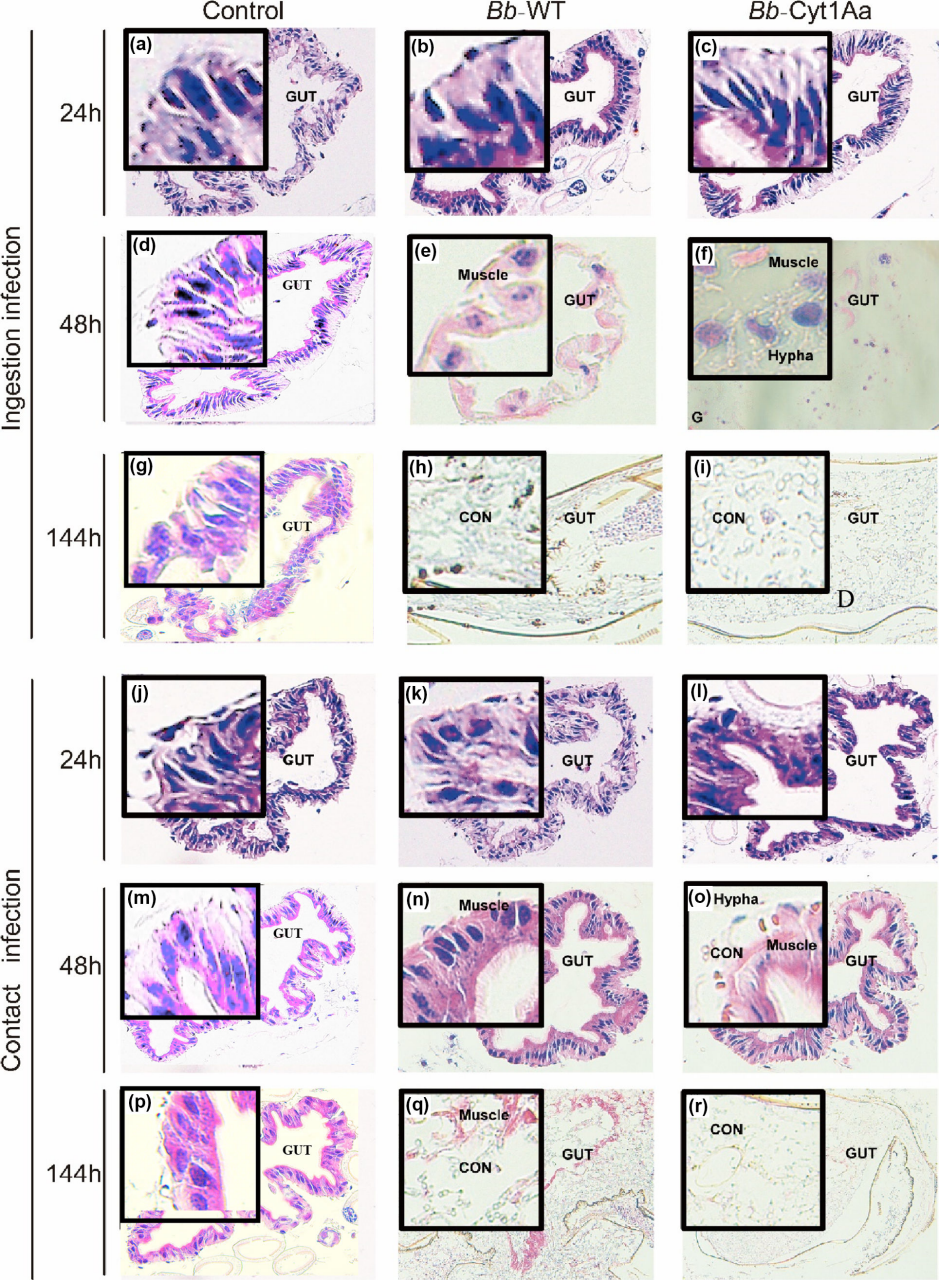
Hyphal growth and distribution in *A. aegypti* midgut infected with *Bb*-Cyt1Aa and WT strain. (a–i) Photomicrography of hyphae and conidia of *Bb*-Cyt1Aa and WT strain infecting *A. aegypti* at 24 h (a–c), 48 h (d–f), or 144 h (g–i) via conidia ingestion. (j–r) Photomicrography of hyphae and conidia of *Bb*-Cyt1Aa and WT infecting *A. aegypti* at 24 h (j–l), 48 h (m–o) or 144 h (p–r) via cuticle contact. Compared to the hyphae of WT, the hyphae of *Bb*-Cyt1Aa showed a significantly higher quantity in the midgut of *A. aegypti* at 48 h, and exhibited a more extensive distribution throughout the midgut of *A. aegypti* at 144 h. The hyphae of *Bb*-Cyt1Aa infecting *A. aegypti* via conidia ingestion were grown better in number compared to direct contact with *Bb*-Cyt1Aa cuticle.

*A aegypti* were infected with *Bb*-Cyt1Aa and *Bb*-WT strains through conidia ingestion or cuticle contact. Following infection, paraffin sections were made of the *A. aegypti* abdomen. Within 24 h of infection, no noticeable differences were observed in the slices of the *A. aegypti* infected with *Bb*-Cyt1Aa and *Bb*-WT strains, whether through conidia ingestion (Fig. 3a through c) or cuticle contact (Fig. 2j through l). However, after 48 h, it was found that the *Bb*-Cyt1Aa strain had more hyphae (thread-like structures) in the midgut of *A. aegypti* infected through conidia ingestion compared to the WT strain (Fig. 3d through f). Similarly, when *A. aegypti* were infected through cuticle contact, more *Bb*-Cyt1Aa hyphae and conidia were observed in the midgut compared to the WT strain (Fig. 3m through o). After 144 h of infection, compared to the *Bb*-WT infection, *A. aegypti* infected with *Bb*-Cyt1Aa through conidia ingestion showed a more extensive distribution of white hyphae, forming a net-like pattern (Fig. 3g through i), and those infected with *Bb*-Cyt1Aa through cuticle contact exhibited more extensive damage to muscle (Fig. 3p and q). *A. aegypti* that ingested glucose solution infected with *Bb*-Cyt1Aa conidia showed a higher quantity of hyphae and more serious damage in the midgut compared to those that had direct contact with *Bb*-Cyt1Aa through their cuticle.

### Increased virulence of *Bb*-Cyt1Aa against *A. albopictus* compared to WT

**FIG 4:**
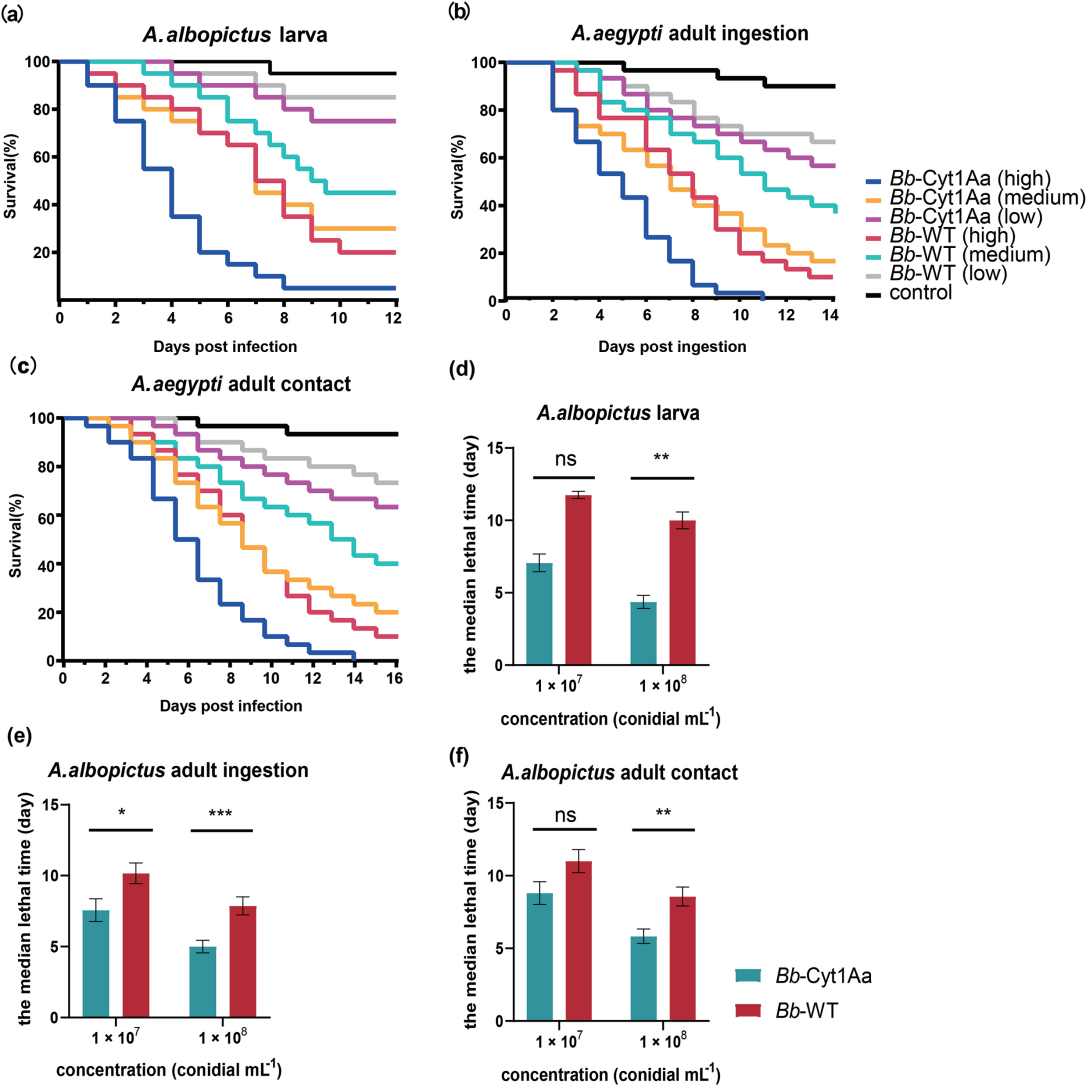
Insecticidal activity of *Bb*-Cyt1Aa and WT strain against *A. albopictus* mosquitoes. (a) The survival curve of *A. albopictus* larvae infected 1 × 10^8^ (high), 1 × 10^7^ (medium), and 1 × 10^6^ (low) conidia mL^−1^ suspensions of *Bb*-Cyt1Aa and WT strain. (b and c) The survival curve of *A. albopictus* adults infected 1 × 10^8^ (high), 1 × 10^7^ (medium), and 1 × 10^6^ (low) conidia mL^−1^ suspensions of *Bb*-Cyt1Aa and WT strain via conidia ingestion (b) or cuticle contact (c). (d) The median lethal times (LT_50_s) of the *Bb*-Cyt1Aa and WT against *A. albopictus* (larvae or adult) at 1 × 10^8^ and 1 × 10^7^ conidia mL^−1^ concentrations. (E and F) The median lethal times (LT_50_s) of the *Bb*-Cyt1Aa and WT against *A. albopictus* adults at 1 × 10^8^ and 1 × 10^7^ conidia mL^−1^ concentrations via conidia ingestion (e) or cuticle contact (f). Data are presented as the median ± SEM (*P < 0.05, **P < 0.01, ***P < 0.001).

In the bioassays with *A. albopictus,* we observed the same phenomenon as we observed in the bioassays with *A. aegypti*. Compared to the WT strain, *Bb*-Cyt1Aa strains significantly reduced the survival of *A. albopictus* (Table 1 ; Fig. 4a through c; Table S1). The LT_50_s of the *Bb*-Cyt1Aa strain were shorter than those of the WT strain (Table 2 ; Fig. 4d through f; Table S2). Compared to the WT strain, the LT_50_ of *Bb*-Cyt1Aa for *A. albopictus* larvae declined by 42.9% at a concentration of 10^8^ conidia/mL and 22.2% at 10^7^ conidia/mL. Compared with the WT, the LT_50_ of *Bb*-Cyt1Aa for *A. albopictus* through conidia ingestion declined by 37.5% at 10^8^ conidia/mL and 36.4% at 10^7^ conidia/mL, and that for *A. albopictus* infection through cuticle contact declined by 37.5% and 33.3% at the same concentrations, respectively. The results demonstrated that *Bb*-Cyt1Aa increased insecticidal activity against *A. albopictus* with 1.29- to 1.75-fold higher toxicity than the WT strain.

## DISCUSSION

Recombinant *B. bassiana* represents an innovative approach to pest control by harnessing the potential of genetic modification (29, 30). It offers the possibility of developing more targeted and efficient biocontrol agents to manage insect pests in various sectors, including agriculture, public health, and forestry (31, 32). It offers several advantages over traditional chemical pesticides, such as being eco-friendly, less harmful to nontarget organisms, and having a lower propensity for developing resistance (33–36). Researchers have been studying the possibility of using recombinant *B. bassiana* as a means of enhancing its efficacy against specific insect species (37–42). Genes-encoding insecticidal toxins derived from scorpions or bacteria have been introduced into *B. bassiana* to make it more lethal to mosquitoes (43–45). By integrating these genes into the fungal genome, recombinant *B. bassiana* can produce and deliver toxins as part of its infection process (46). When the fungus comes into contact with mosquitoes, toxins are released and can cause damage to the mosquito digestive system or other vital organs, ultimately leading to death (47).

Cyt1Aa is a toxin produced by *B. thuringiensis* that can be used for mosquito control (48, 49). A study showed that mosquitoes treated with Cyt1Aa exhibited a faster rate of mortality than control mosquitoes (50). The receptor for Cyt1Aa is present in the midgut of mosquitoes and does not interact with other beneficial organisms’ cells (51, 52). This made it highly specific to mosquitoes. In our study, *B. bassiana* was modified to express Cyt1Aa to increase its virulence against *A. aegypti* and *A. albopictus*. The *Cyt1Aa* gene was stably inherited in the *Bb*-Cyt1Aa strain. *Bb*-Cyt1Aa significantly reduced the survival of *A. aegypti* and *A. albopictus* compared to the *Bb*-WT strain.

The expression of Cyt1Aa toxins allows *B. bassiana* to target the midgut epithelial cells of mosquitoes (53). This targeted approach contributed to a quicker mortality rate. When the *Bb*-Cyt1Aa strain infects mosquitoes, it colonizes and uses various mechanisms to evade the mosquito’s immune system (47). As part of this infection process, the fungus produces and secretes Cyt1Aa toxins (54). Cyt1Aa acts by binding to specific receptors on the membrane of midgut epithelial cells (55). This binding triggers a conformational change in Cyt1Aa, allowing it to insert into the cell membrane and form pores or channels (56, 57). These pores disrupt the integrity of the midgut membrane, leading to the leakage of cellular contents and loss of osmotic balance. In our study, we prepared paraffin sections of infected mosquitoes and observed tissue changes. Within 48 h of infection, compared with the WT strain, we observed the presence of conidia in the midgut of mosquitoes infected with the *Bb*-Cyt1Aa strain. Meanwhile, at 48 h, we observed that partial cell membrane damage occurred in the tissue infected with *Bb*-Cyt1Aa through conidium ingestion. Cyt1Aa may aid in the colonization process by helping the fungus disrupt cell membranes to potentially facilitate nutrient acquisition for the growing fungus. Compared to direct contact with *B. bassiana* spores, a higher quantity of conidia was observed in the midgut when the mosquito consumed glucose solution contaminated with *B. bassiana* conidia. The observed difference in conidia presence between the two experimental conditions may be related to the mode of entry into the mosquito’s midgut. When the mosquito ingests contaminated glucose solution, more spores may be able to reach and colonize the midgut (58). After 6 days of infection, we observed the leakage of cellular contents and the growth of white hyphae throughout the entire tissue.

*B. bassiana* hyphae can penetrate an insect’s exoskeleton through two main mechanisms: superficial growth and invasion of the body (59). In superficial growth, the *B. bassiana* hyphae grow and spread on the outside of the mosquito’s exoskeleton (60). They may form a visible white coating on the mosquito’s body. Once inside, the hyphae of *B. bassiana* can spread and colonize various tissues of the mosquito, causing damage and potentially leading to the death of the insect (61, 62). In our study, after being infected for 6 days with the *Bb*-Cyt1Aa and *Bb*-WT strains through cuticle contact, the surface of the dead mosquitoes displayed white hyphal growth. *Bb*-Cyt1Aa hyphae grew faster and stronger than those of the WT strain. For mosquitoes infected with *Bb*-Cyt1Aa for 6 days, compared with the WT strain, the paraffin sections displayed a more extensive distribution of white hyphae forming a net-like pattern. The *Bb*-Cyt1Aa strain has been shown to cause more extensive damage to mosquitoes, particularly through ingestion-based infection. Through midgut targeting, membrane disruption, subsequent cell lysis, and hyphal growth, *B. bassiana* expressing Cyt1Aa effectively kills mosquitoes, enhancing its virulence against *Aedes* mosquitoes.

In our bioassays, we only used three concentration groups to compare the virulence of the *Bb*-Cyt1Aa strain with the WT strain. This uncertainty arises from the possibility that the chosen concentrations do not encompass the full range of conditions in the natural environment. The observed virulence in the bioassays could be potentially underestimated when compared to the *Bb*-Cyt1Aa strain’s capabilities under optimal conditions. Furthermore, due to the limited testing concentration, it is also impossible to accurately calculate the credible median lethal concentration, which unveils the precise concentration required to induce mosquito mortality (43, 63). Therefore, future studies should aim to include a broader range of concentrations to obtain a more precise understanding of the *Bb*-Cyt1Aa strain’s genuine potential for virulence enhancement compared to the WT strain.

Apart from the multitude of limitations that need to be addressed in future research, our study highlights several noteworthy aspects. One such aspect is the employment of an efficient method in constructing a recombinant *B. bassiana* strain. By selectively inserting specific exogenous toxin genes, such as Cyt1Aa, into the genome of the environmentally friendly fungus *B. bassiana*, we successfully created the recombined *B. bassiana* strain *Bb*-Cyt1Aa, which possesses enhanced toxicity against mosquitoes compared to the WT strain (64–66). This level of specificity and customization allows for the design of tailored biocontrol strategies, reducing environmental impact by minimizing the use of chemical inputs and increasing the success rate of specific pest control efforts (67). In the future, we will optimize the expression of exogenous toxins, understand their potential side effects, and evaluate their long-term impact on nontarget organisms.

### Conclusions

Our findings suggest that the expression of the *B. thuringiensis* toxin Cyt1Aa in *B. bassiana* has enhanced its virulence against *A. aegypti* and *A. albopictus* mosquitoes, making it more efficient in eradicating these species. This novel strain can be employed in conjunction with chemical insecticides to decrease the dependence on harmful substances, consequently mitigating adverse effects on the environment and human health.

## SUPPLEMENTAL MATERIAL

Supplemental tables spectrum.03792-23-s0001.docx Tables S1 and S2.

ASM does not own the copyrights to Supplemental Material that may be linked to, or accessed through, an article. The authors have granted ASM a non-exclusive, world-wide license to publish the Supplemental Material files. Please contact the corresponding author directly for reuse.
