# Supplementary material for: Recombinant Beauveria bassiana expressing Bacillus thuringiensis toxin Cyt1Aa: a promising approach for enhancing Aedes mosquito control
Source: Microbiol Spectr. 2024 May 29;12(7):e03792-23. doi: 10.1128/spectrum.03792-23 (PMC11218515; doi:10.1128/spectrum.03792-23)
Supplement: Supplemental tables — Tables S1 and S2. [file spectrum.03792-23-s0001.docx]

**Table S1** Results of the log-rank test for the different concentrations of *Bb*-Cyt1Aa or the WT against *Ae. aegypti* or *Ae. albopictus* mosquitoes

| **Mosquitoes** | **Fungal strains** | **χ^2^** | **Df** | ***P*** |
| --- | --- | --- | --- | --- |
| *Ae. aegypti* larvae | *Bb*-Cyt1Aa | 33.683 | 2 | <0.001 |
|  | WT | 16.023 | 2 | <0.001 |
| *Ae. aegypti* adults (ingestion) | *Bb*-Cyt1Aa | 46.037 | 2 | <0.001 |
|  | WT | 28.123 | 2 | <0.001 |
| *Ae. aegypti* adults  (contact) | *Bb*-Cyt1Aa | 43.314 | 2 | <0.001 |
|  | WT | 24.435 | 2 | <0.001 |
| *Ae. albopictus* larvae | *Bb*-Cyt1Aa | 31.913 | 2 | <0.001 |
|  | WT | 16.627 | 2 | <0.001 |
| *Ae. albopictus* adults  (ingestion) | *Bb*-Cyt1Aa | 37.824 | 2 | <0.001 |
|  | WT | 21.566 | 2 | <0.001 |
| *Ae. albopictus* adults  (contact) | *Bb*-Cyt1Aa | 42.279 | 2 | <0.001 |
|  | WT | 27.756 | 2 | <0.001 |

*P* < 0.05 means that the difference is significant.

**Table S2** Results of the log-rank test on the *Bb*-Cyt1Aa and WT strain against *Aedes aegypti* or *Aedes albopictus* (larvae or adults) at each given concentration.

| **Mosquitoes** | **Concentrations (conidia/ml)** | **χ^2^** | **Df** | ***P*** |
| --- | --- | --- | --- | --- |
| *Ae. aegypti* larvae | 1×10^8^ | 7.283 | 1 | 0.007 |
|  | 1×10^7^ | 0.893 | 1 | 0.345* |
|  | 1×10^6^ | 0.329 | 1 | 0.566* |
| *Ae. albopictus* larvae | 1×10^8^ | 9.208 | 1 | 0.002 |
|  | 1×10^7^ | 1.421 | 1 | 0.233* |
|  | 1×10^6^ | 0.586 | 1 | 0.444* |
| *Ae. aegypti*  adults ingestion | 1×10^8^ | 14.127 | 1 | <0.001 |
|  | 1×10^7^ | 4.242 | 1 | 0.039 |
|  | 1×10^6^ | 0.682 | 1 | 0.409* |
| *Ae. albopictus*  adults ingestion | 1×10^8^ | 13.497 | 1 | <0.001 |
|  | 1×10^7^ | 4.765 | 1 | 0.029 |
|  | 1×10^6^ | 0.573 | 1 | 0.449* |
| *Ae. aegypti*  adults contact | 1×10^8^ | 12.099 | 1 | 0.001 |
|  | 1×10^7^ | 3.028 | 1 | 0.082* |
|  | 1×10^6^ | 0.276 | 1 | 0.600* |
| *Ae. albopictus*  adults contact | 1×10^8^ | 10.100 | 1 | 0.001 |
|  | 1×10^7^ | 3.756 | 1 | 0.053* |
|  | 1×10^6^ | 0.734 | 1 | 0.392* |

*P*<0.05 means that the difference is significant.

* indicates that there is no statistical significance in the mortality rates between the *Bb*-Cyt1Aa group and the *Bb*-WT group.
